# Comparative Effects of Conventional and Systemic Biologicals on the Development and Yield of Two Carrot Cultivars

**DOI:** 10.3390/microorganisms14081834

**Published:** 2026-08-19

**Authors:** Gerardo Armando Aguado-Santacruz, Glenda Margarita Gutiérrez-Benicio, Cesar Leobardo Aguirre-Mancilla, Jesús Manuel Arreola-Tostado, Mónica Guadalupe Lozano-Contreras

**Affiliations:** 1 Campo Experimental Bajío, Instituto Nacional de Investigaciones Forestales, Agrícolas y Pecuarias, km 6.5 Carretera Celaya-San Miguel de Allende, Celaya 38110, Guanajuato, Mexico; 2 BIOqualitum, Oriente 7 # 158, Ciudad Industrial, Celaya 38010, Guanajuato, Mexico; manuelito1876@gmail.com; 3 Tecnológico Nacional de México/IT de Roque, km 8 Carretera Celaya–Juventino Rosas, Celaya 38110, Guanajuato, Mexico; glenda.gb@roque.tecnm.mx (G.M.G.-B.); cesar.am@roque.tecnm.mx (C.L.A.-M.); 4 Campo Experimental Mocochá, Instituto Nacional de Investigaciones Forestales, Agrícolas y Pecuarias, Antigua Carretera Mérida-Motul km 24.5, Mocochá 97454, Yucatán, Mexico

**Keywords:** biofertilizer, bionematicide, second-generation biologicals, stomata, endophyte, plant growth-promoting microorganisms, *Azospirillum brasilense*, *Pseudomonas fluorescens*, *Bacillus subtilis*, *Bacillus mojavensis*, *Paecilomyces lilacinus*, *Pochonia chlamydosporia*

## Abstract

Systemic biologicals have emerged as a disruptive technology for overcoming the limitations of conventional biologicals. The effectiveness of systemic and conventional biological products in improving the growth and yield of two carrot cultivars was compared. Carrot plants were inoculated with either systemic or conventional biofertilizers and nematicides, and various growth parameters and yields were compared. As expected, the first indicator of the proper functioning of the systemic biofertilizer was increased chlorophyll content (two-variety averages: systemic = 17.1, conventional = 11.8, control = 10.9 mg g^−1^ FW). The results indicated the superior performance of the systemic biologicals in carrot production. The two-variety average yield increased by 11.92 t ha^−1^ (32%) in plants inoculated with systemic biologicals compared with the control plants and by 6.62 t ha^−1^ (15.4%) compared with plants inoculated with conventional products (*p* ≤ 0.05). For the biological nematicides, the two-variety average damage rate was 10.15% in plants treated with the systemic bionematicide, 21.15% in plants treated with the conventional bionematicide, and 31.6% in non-inoculated plants. Analyses revealed the presence of microorganisms in plants inoculated with systemic biologicals, although beneficial fungi were detected at relatively low levels. In conclusion, systemic products offer a more robust and consistent technology for improving crop health and yield and are considered the next generation of biologicals.

## 1. Introduction

Carrot (*Daucus carota* L.) is a widely grown and consumed vegetable crop worldwide. Botanically, carrot belongs to the Apiaceae family, has high nutritional value, provides numerous health benefits, and is of considerable economic relevance. Carrots are rich in valuable compounds, including carotenoids, anthocyanins, dietary fiber, vitamins, and other essential nutrients. They play important roles in human nutrition and serve as raw materials for the nutraceutical, food, and pharmaceutical industries [1].

Originally from Central Asia, particularly regions near Afghanistan [2], carrots later spread to the Mediterranean area [3]. Today, they rank among the ten most widely produced vegetable crops worldwide. As of 2022, the leading producers of carrots were China (18.7 million tons), Uzbekistan (3.9 million tons), and the United States (1.4 million tons) [4].

Despite the growing global emphasis on reducing chemical inputs in agriculture, global statistics do not indicate a substantial decline in their use. Over a 22-year period, the application of inorganic fertilizers in agriculture rose by 37%, increasing from 135 million tons in 2000 to 185 million tons of nutrients in 2022. This growth included a 33% increase in nitrogen use, a 29% increase in phosphorus, and a 63% increase in potassium. The largest consumers of inorganic fertilizers, in descending order, were China, India, Brazil, and the United States [5].

The use of pesticides increased by 70% between 2000 and 2022 (from 2.2 to 3.7 million tons). The largest shares of global pesticide use occurred in the Americas, followed by Asia, Europe, Africa, and Oceania. The share of global pesticide use in the Americas, which remains the leading contributor, has increased from 41% to 51%, while Asia and Europe have experienced slight declines, from 33% to 29% and from 21% to 13%, respectively. In 2021, Brazil was the top pesticide consumer, with 0.8 million tons, accounting for 22% of global usage, well ahead of the United States (0.47 million tons) and Indonesia (0.29 million tons). During 2022, pesticide application rates were highest in the Americas (5.1 kg ha^−1^), followed by Oceania, Asia, Europe, and Africa. Notably, Asia and Europe were the only regions where pesticide use per unit of cropland slightly declined between the early 2010s and 2022, decreasing from 2 to 1.8 kg ha^−1^ and from 1.7 to 1.5 kg ha^−1^, respectively [4].

Like all agricultural crops, carrot yield and quality depend on numerous factors, including soil fertility and irrigation. Unfavorable growing conditions result in physiological anomalies and reduced root production and quality, thereby reducing market value or resulting in losses of up to 30% [6]. In addition, carrot plants are susceptible to numerous diseases and pests, reducing their profitability [1]. The carrot crop is affected by more than 150 species of insects, mites, nematodes, fungi, viruses, bacteria, and phytoplasmas. Among these, pests such as nematodes and carrot flies, as well as diseases such as black rot, cavity spot, crown rot, cottony rot, and leaf blight, have emerged as major challenges worldwide [7].

Although inoculation with plant growth-promoting microorganisms (PGPMs) is a promising technology for improving the yield and quality of carrot and other crops worldwide and for reducing the impact of agrochemicals on the environment, the consistency and reliability of these products remain challenges to be addressed [8,9], under the assumption that these products are manufactured in compliance with basic quality standards. A study carried out in Mexico in 2012, in which the 25 best-selling biological products in agriculture were evaluated, revealed that 90% of the products analyzed did not meet basic quality standards [10].

PGPR inoculation offers numerous benefits for crops, including enhanced germination and seedling development, increased root system development, enhanced plant health and uniformity, improved seedling establishment, increased chlorophyll accumulation, reduced lodging, shorter harvest times, and extended and increased productivity of perennial and greenhouse crops [11].

Taking into account the limitations of conventional biological products, in 2010, our research group developed an innovative and disruptive technology called “Micro In.” This technology allows beneficial microorganisms to be introduced into plants through the stomata, making their use in agriculture a more efficient and reliable tool for enhancing crop productivity, health, and quality. Products derived from this technology are known as “systemic biological products” and offer several advantages over conventional biological products [12]. Compared with the soil environment, the internal plant tissue structure offers a more conducive habitat for the survival and multiplication of beneficial microorganisms, enhancing plant growth. Furthermore, these microorganisms operate more effectively and reliably since the bioactive compounds that support plant development and wellness are delivered directly within the plant system, function more directly, and avoid potential losses [13]. Systemic biologicals are the only products that can be used in hydroponic agriculture, for mangrove restoration, for combating microbial systemic diseases such as ‘Huanglongbing’, and for the inoculation of biological products via drone technology. Additional advantages of systemic/endophytic biologicals have been discussed by other authors [11,14,15].

Existing studies on PGPR inoculation or co-inoculation in carrot plants are scarce and often evaluate only a limited number of parameters, lack bacterial species identification, evaluate only part of the crop cycle, or fail to examine interactions under reduced fertilizer application conditions [16].

Various authors have found that carrot root biomass can be increased by certain bacterial strains, such as *Bacillus subtilis*, *B. methylotrophicus* and *Streptomyces griseus* [17,18,19]. Conversely, Matsuoka et al. [20] evaluated the effects of three ACC deaminase-producing bacteria on the growth of carrot plants, and no significant differences were found between two of them and the control, whereas the third strain significantly reduced the growth of carrot roots.

Recently, the benefits of emerging systemic biological products have been proven in sugarcane, onion, and other crops [11,21,22]. However, the effectiveness of conventional and systemic biological products under field conditions has not been compared. This comparison is important for guiding future research focused on improving the performance and consistency of biological products in the field. Therefore, the objective of this research was to compare the effectiveness of conventional and systemic biological products in improving the growth, quality, and yield of two carrot varieties.

## 2. Materials and Methods

### 2.1. Study Site and Climatic Conditions

This research was conducted from August to November 2021 in San Juan de la Vega, Municipality of Celaya, Guanajuato, Mexico (20°38′22.2″ N; 100°45′34.5″ W), at an altitude of 1750 m.a.s.l. The soils at this site are classified as Vertisols [23], which are dark, fertile soils with a high organic matter content. The carrot varieties evaluated were ‘Romance’ (Nunhems/BASF, Nunhem, The Netherlands) and ‘Baltimore’ (Bejo Seeds, Inc., Oceano, CA, USA). Three hectares of each carrot variety were sown at a seeding rate of 350,000 plants per hectare. The bed width was 118 cm, and carrots were sown in twin rows using two drip lines. The spacing between rows was 28 cm, and plants were spaced 8 cm apart within each row.

**Figure 1:**
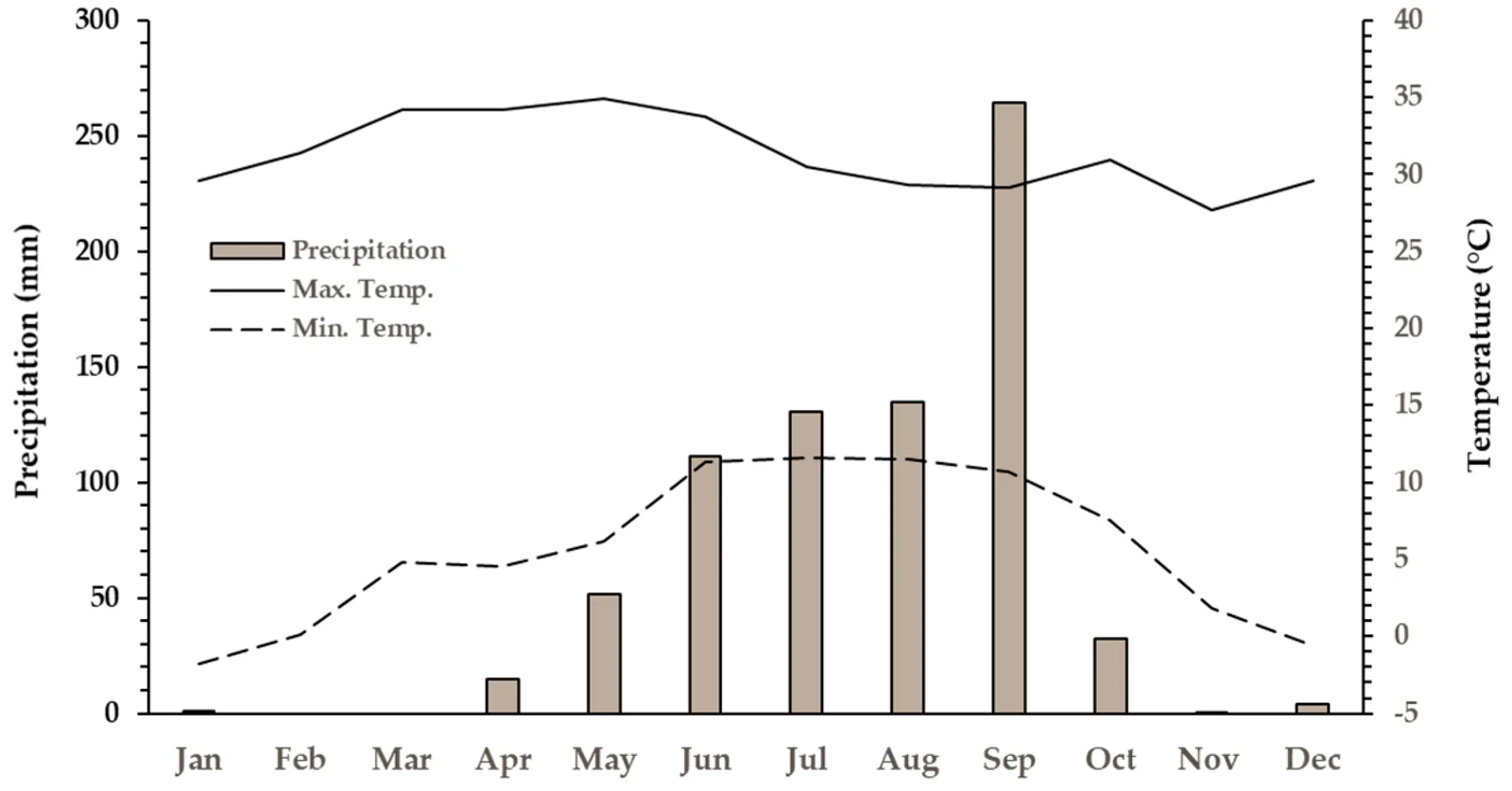
Climograph of San Juan de la Vega, Guanajuato, Mexico, showing the precipitation and the maximum and minimum temperatures during 2021 (prepared using data from FGP, 2021).

The health and yield of carrot plants were compared across three treatments: (a) plants inoculated with conventional biological products, (b) plants treated with systemic biological products, and (c) plants managed conventionally without biological products (control). The biological treatments consisted of a mixture of a biofertilizer and a bionematicide, either systemic or conventional. The experiment followed a completely randomized design comprising three treatments and six replicates. In 2021, the annual rainfall was 745.6 mm (432.4 mm during the carrot growing cycle), and the annual maximum and minimum temperatures were 31.3 °C and 5.6 °C, respectively. During the carrot growing cycle in the experiment, these temperatures were 29.2 °C and 7.9 °C, respectively (Figure 1). Finally, the mean relative humidity and total evaporation during the productive cycle were 69.5% and 444.0 mm, respectively [24].

### 2.2. Experimental Treatments

This research compared the effectiveness of two biological treatments using either conventional or systemic products. The conventional biological treatment involved microorganisms from the microbial collection of BIOqualitum (www.bioqualitum.com): a biofertilizer containing *Pseudomonas fluorescens*, *Azospirillum brasilense*, and *Bacillus subtilis*, and a bionematicide containing *Paecilomyces lilacinus*, *Pochonia chlamydosporia*, and *Bacillus mojavensis*. The systemic biological treatment also involved products from BIOqualitum (Celaya, Mexico) (Patent 328184) with the capacity for systemic transport within plants: the biofertilizer BactoCROP^®^ (BIOqualitum S.A. de C.V., Celaya, Mexico) and the bionematicide NemaCRACK^®^ (BIOqualitum S.A. de C.V., Celaya, Mexico). The systemic biofertilizer contained *Pseudomonas fluorescens* Ag_001, *Azospirillum brasilense* Ag_001, and *Bacillus subtilis* Ag_001. The systemic bionematicide contained *Paecilomyces lilacinus* Ag_001, *Pochonia chlamydosporia* Ag_001, and *Bacillus mojavensis* B3-15. Both conventional and systemic biological products were formulated using the same vehicle components (Table 1) and reached a minimum concentration of 1 × 10^8^ CFU of each microorganism.

The application of both products was integrated into the local agronomic management practices for carrot production. They were applied during the 2021 growing cycle of the two carrot varieties, with three applications of conventional or systemic biological products made to the one-hectare experimental plots.

Chemical fertilization was adjusted to 150–80–150 (NPK) in all evaluated treatments using ammonium sulfate, triple calcium superphosphate, and potassium chloride. Nitrogen was applied at three stages: seeding, 30 days after seeding, and 60 days after seeding; all P and K were applied at seeding. When carrot plants reached the 5- to 8-leaf stage, they were foliar-sprayed with micronutrient-containing fertilizers supplying boron (2 kg ha^−1^), zinc (5 kg ha^−1^), copper (3 kg ha^−1^), and manganese (3 kg ha^−1^).

To control weeds, a mixture of the herbicides linuron and fluazifop-p-butyl was applied at 25 and 45 days after seeding at doses of 940 and 250 g ha^−1^, respectively. A final manual weed removal was performed at 75 days after seeding. Foliar diseases were controlled through applications of elemental sulfur and copper oxychloride at doses of 1370 and 15 g ha^−1^, respectively. These applications were performed 45 days after seeding and coincided with each irrigation event. As stated, conventional and systemic biological products were applied to the experimental plots in addition to the chemical products normally employed by local farmers; however, 1,3-dichloropropene, a nematicide commonly used by local farmers, was not applied to the biologically treated plots. The three applications of the conventional and systemic biological products were performed 15, 45, and 75 days after seeding. During the first application, 1.5 kg of each biofertilizer and 500 g of each bionematicide were dissolved together in 600 L of water and applied to the respective one-hectare carrot plots using a tractor sprayer. During the second and third applications, 2.0 kg of each biofertilizer and 750 g of each bionematicide were injected into the drip irrigation system. In addition, 1 kg of each biofertilizer and 250 g of each bionematicide were dissolved in 400 L of water and foliar-sprayed onto the experimental carrot plots. A neighboring plot of the same size was managed conventionally, according to the complete chemical product application program normally employed by local farmers, including chemical fertilization and the use of chemical pesticides.

### 2.3. Variables Analyzed

The chlorophyll content of carrot leaves [25] and nematode root damage were evaluated 45 and 90 days after seeding, respectively, using 45 plants and 150 roots randomly collected from each treatment/variety. In the evaluation of nematode root damage, forked, stubby, or malformed roots were identified as being infected with nematodes [26]. The population density of nematodes (*Meloidogyne incognita*) quantified in the soil of the experimental areas was 475 J2 per 200 g of soil.

At the end of the growing cycle (120 days after seeding), the root diameter, plant length, root weight, and number of plant leaves were measured for the different treatments/varieties, with 100 plants randomly selected from each treatment. Finally, to determine carrot yield, ten randomly selected plots were harvested per treatment from each variety parcel; each plot consisted of five rows of carrots (15 m long and 28 cm row spacing).

### 2.4. Statistical Analysis

One-way ANOVA was used to analyze differences in the total chlorophyll content, root diameter, plant length, number of leaves, and root weight, as well as in the total carrot yield, among the different treatments and varieties. Significant differences (*p* ≤ 0.05) between means were determined via Tukey’s test [27]. Statistical differences (*p* ≤ 0.05) in the percentage of nematode-damaged carrot plants among the treatment groups were analyzed via Fisher’s exact test [28].

### 2.5. Detection of the Presence of Beneficial Microorganisms Within Internal Tissues of Carrots

At the end of the carrot growing cycle, 35 plants from each of the two biologically treated plots and from the conventionally managed parcel were randomly collected to reisolate the bacteria and fungi previously applied to the carrot plants. The root samples were subsequently washed under running water and cut into small fragments (2 × 2 cm). These pieces were subsequently disinfected with 70% ethanol for 1 min and 3% sodium hypochlorite for 2 min, rinsed three times with sterile distilled water and then placed on sterile paper napkins to eliminate excess moisture. Finally, the carrot fragments were cut into smaller fragments (1 × 1 cm) and placed in Petri dishes containing selective media for the isolation of *Bacillus subtilis* (BS medium) [29], *Azospirillum brasilense* (Congo Red and Elmarc media [30], *Pseudomonas fluorescens* (Gould S1 medium [31], *Bacillus mojavensis* (TSA medium [32], and *Paecilomyces lilacinus* (PDA supplemented with chloramphenicol and sodium chloride [33]) and *Pochonia chlamydosporia* (water agar supplemented with chlortetracycline-HCl and streptomycin sulfate [34]). The Petri dishes containing the carrot pieces were incubated for 24 h at 28–30 °C. Morphologically distinct microbial colonies were selected and purified. The *Paecilomyces lilacinus* and *Pochonia chlamydosporia* strains were analyzed under a microscope to measure the size and morphology of the conidia, conidiophores, mycelia, and other important fungal structures, using taxonomic identification keys specifically developed by our scientific group to identify and distinguish our fungal strains. For example, the identification of *P. chlamydosporia* was based on the abundance and location of dictyochlamydospores, conidiophores, and whether the conidia were borne in chains or heads [35]. Finally, molecular analyses were carried out to identify the specific bacterial strains inoculated on the carrot plants.

### 2.6. DNA Extraction, PCR, and Restriction Analysis for Bacterial Strains

In brief, the reisolated microorganisms were cultured in 10 mL of their respective media for 24 h. The cultures were then centrifuged at 5000 rpm for 10 min, after which the supernatant was removed, and the resulting pellets were used for genomic DNA extraction using the sarcosine method [36]. DNA quality was assessed by electrophoresis on 1% agarose gels.

The extracted DNA served as a template to amplify fragments of the internal transcribed spacer (ITS) regions using the primers G1 (SEC ID NO:7) and L1 (SEC ID NO:8) [37]. Each 50 μL reaction mixture contained 150 ng of genomic DNA, 1 X buffer, 2 mM MgCl_2_, 50 μM of each nucleotide, 0.2 μM of each primer, and 1 unit of Taq polymerase. Amplification was carried out in a Thermo Scientific thermocycler with the following program: an initial step of 5 min at 94 °C; 35 cycles consisting of 1 min at 94 °C, 2 min at 55 °C, and 2 min at 72 °C; and a final extension of 7 min at 72 °C. The samples were then held at 4 °C.

The resulting amplicons were purified using a QIAEX II kit (Qiagen, Hilden, Germany) and subsequently digested with the DdeI enzyme (Table 2). Previously, DNA fingerprinting of three bacterial strains was performed on the basis of restriction patterns of ITS fragments generated using the enzymes HaeIII, DdeI, and HhaI.

## 3. Results

The climatic conditions during the field evaluation of the biological products were favorable for carrot growth and development. Precipitation during the carrot growing cycle (432.4 mm) was within the range considered suitable for carrot growth. The maximum and minimum temperatures recorded from August to November (29.2 °C and 7.9 °C, respectively) were also favorable for carrot growth (Figure 1).

The first indicator of the effectiveness of the systemic biofertilizer was an increase in leaf chlorophyll content. Inoculation with the systemic products increased the leaf chlorophyll content by 50% and 38.5% in the ’Romance’ and ’Baltimore’ varieties, respectively, compared with inoculation with the conventional biofertilizer (*p* ≤ 0.05; Table 3). The increases were even greater when the systemic biofertilizer was compared with the control, reaching 60% in ’Romance’ and 50% in ’Baltimore’ (*p* ≤ 0.05). In contrast, leaf chlorophyll content did not differ significantly between plants inoculated with the conventional biofertilizer and the non-inoculated control plants (*p* ≥ 0.05; Table 3).

By the end of the carrot growing cycle, additional effects of the systemic biological products were evident in the measured growth parameters of the carrot plants.

Thus, root diameter, plant length, number of leaves, and root weight were significantly greater in ‘Romance’ plants inoculated with systemic biological products (*p* ≤ 0.05) than in those inoculated with conventional biological products or in the non-inoculated control plants (Table 4). Compared with the control, ‘Romance’ plants inoculated with conventional biological products exhibited greater root diameter and root weight (*p* ≤ 0.05), whereas number of leaves and plant length did not differ significantly between these treatments (*p* ≥ 0.05). Similarly, in the ‘Baltimore’ variety, plants inoculated with conventional or systemic biological products exhibited greater values for all measured growth parameters than the control plants (*p* ≤ 0.05), except for plant length.

Similarly, carrot yield was greater in plants inoculated with conventional or systemic biological products than in non-inoculated plants. Compared with the control, conventional biological products increased carrot yield by 15.6% in ‘Romance’ and 12.5% in ‘Baltimore’ (43.2 vs. 37.4 t ha^−1^ for ‘Romance’ and 42.8 vs. 38.0 t ha^−1^ for ‘Baltimore’; *p* ≤ 0.05; Table 4). However, compared with the control treatment, the systemic biological treatment produced an even greater increase in carrot yield, reaching 31.3% in ‘Romance’ (49.1 vs. 37.4 t ha^−1^; *p* ≤ 0.05) and 31.9% in ‘Baltimore’ (50.16 vs. 38.03 t ha^−1^; *p* ≤ 0.05).

**Figure 2:**
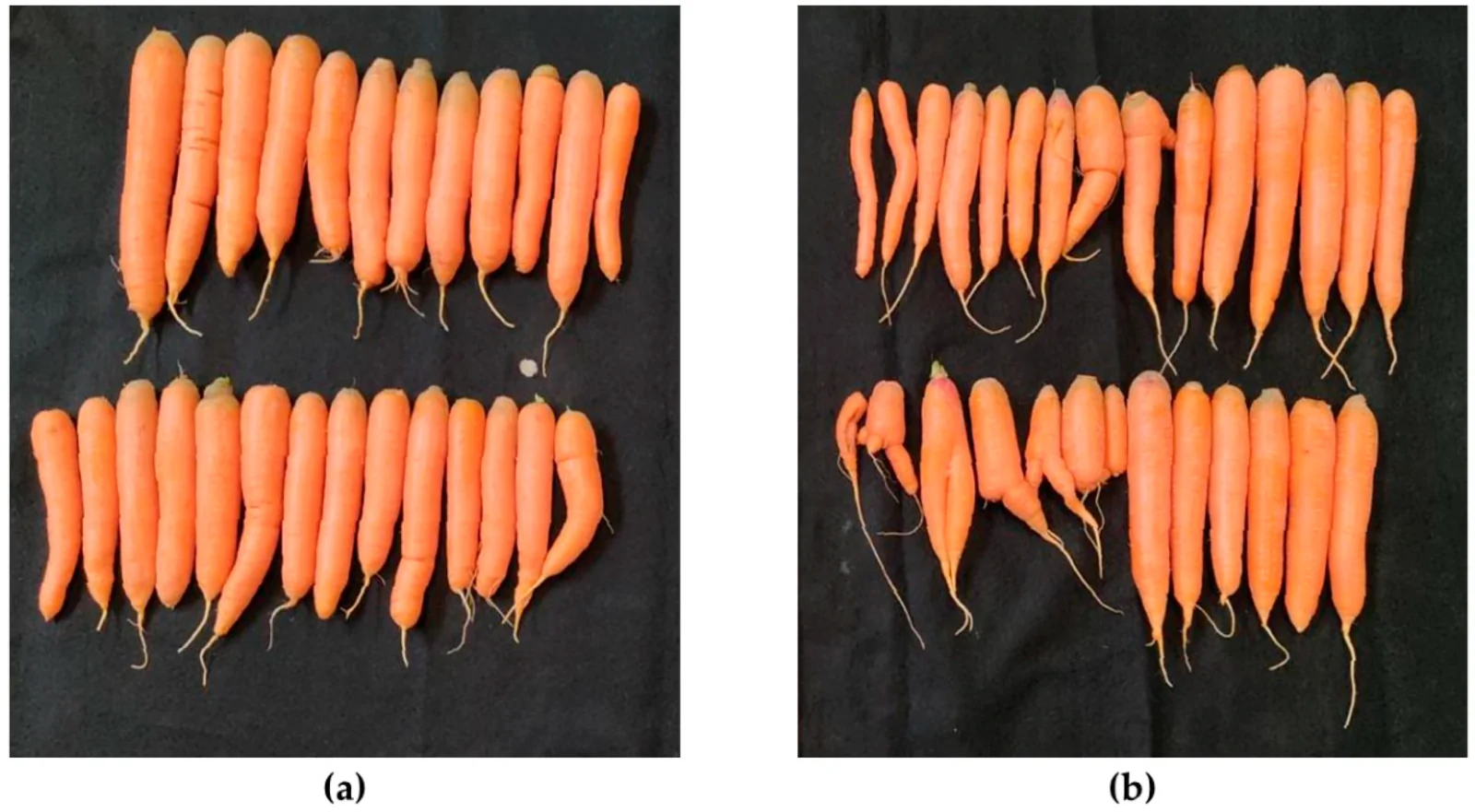
Nematode damage 90 days after seeding in the ‘Romance’ variety. (**a**) Carrots inoculated with systemic biological products and (**b**) non-inoculated control carrots. Carrots damaged by nematodes exhibited developmental abnormalities.

The percentage of carrot roots damaged by nematodes was significantly lower (*p* ≤ 0.05) in both varieties treated with conventional or systemic biological products than in the non-inoculated control plants (Table 5; Figure 2). Furthermore, plants treated with systemic biologicals exhibited significantly less nematode damage (*p* ≤ 0.05) than those treated with conventional biologicals (22.1% vs. 11.0% in ‘Romance’ and 20.2% vs. 9.3% in ‘Baltimore’).

Finally, microbiological and molecular analyses confirmed the presence of microorganisms inoculated through the systemic biological biofertilizer (*Pseudomonas fluorescens*, *Azospirillum brasilense*, and *Bacillus subtilis*) and the systemic biological nematicide (*Bacillus mojavensis*, *Paecilomyces lilacinus*, and *Pochonia chlamydosporia*) within the internal tissues of carrot plants but not in plants treated with conventional biological products (Table 5). None of these microorganisms was detected in the non-inoculated control plants.

The colonization of internal carrot tissues by bacteria included in the systemic biological products ranged from 90 to 95% in the ‘Romance’ variety and from 86 to 90% in the ‘Baltimore’ variety (Table 6). Internal colonization by the fungal components of the systemic biological products ranged from 38 to 45% in both varieties (mean = 42%). In contrast, the microorganisms included in the conventional biological products were not detected within the internal tissues of carrot plants.

## 4. Discussion

Given current trends aimed at developing technologies that reduce the use of agrochemicals in agriculture, the application of organic fertilizers and biological products has received increasing attention. The appeal of this approach lies in its potential to achieve yields equal to or even greater than those obtained with conventional technology while avoiding or reducing the adverse effects associated with agrochemical use, such as environmental contamination and the production of chemically contaminated fruits and vegetables.

As is typical of systemic biological products containing diazotrophic bacteria, the first indication of their effective functioning in plants is an increase in chlorophyll content [21,22,38]. Although increases in chlorophyll and nitrogen contents following inoculation with conventional [39] or systemic biological [11,21] products containing diazotrophic bacteria have been reported previously, this response appears to be more consistent with systemic biological products and may therefore serve as a reliable indicator of the successful establishment and functioning of the inoculants [22]. Other treatments unrelated to the use of diazotrophic bacteria, such as formulations containing organic matter (N 9%, P 1%, and K 4% [38]), microalgae such as *Asterarcys quadricellulare* [40], and foliar organic fertilizers containing micronutrients and hormones [41], can also induce increases in chlorophyll content.

Following this increase in chlorophyll content, improvements in the growth parameters and yield measured in the present study were observed. The results demonstrate the advantages of systemic biological products over conventional biological products in improving carrot performance.

In general, carrot plants treated with systemic biological products exhibited superior performance to both non-inoculated plants and those inoculated with conventional biological products. The differences observed in the growth parameters evaluated in this study indicate that systemic biological products can significantly increase carrot yield.

Numerous studies have shown that carrot plants inoculated with plant growth-promoting microorganisms perform better than those inoculated with organic or chemical nutritional sources alone.

Zdravkovska et al. [42] studied the effects of two types of microbial fertilizers on the morphological characteristics and yield of carrot plants. The treatments were as follows: (a) a control without microbial fertilizer; (b) Variant 1, a microbiological fertilizer containing *Azotobacter*, nitrifying microorganisms, and phosphate-solubilizing microorganisms; and (c) Variant 2, a microbiological fertilizer containing *Azotobacter*, nitrifying microorganisms, phosphate-solubilizing microorganisms, and iron. Measurements of carrot morphological characteristics revealed that (1) whole-plant weight ranged from 73.17 g in the control to 84.00 g in Variant 1; (2) root weight ranged from 63.83 g in the control to 74.67 g in Variant 1; (3) root length ranged from 17.69 cm in the control to 18.28 cm in Variant 1; (4) root width ranged from 2.22 cm in the control to 2.39 cm in Variant 1; and (5) the root index (ratio root length to root width) ranged from 7.65 cm in Variant 1 to 7.99 cm in the control. Significant differences in whole-plant and root weights were observed between Variants 1 and 2. The highest yield was obtained in Variant 1 (53.77 t ha^−1^), whereas yields were 46.32 t ha^−1^ in Variant 2 and 45.96 t ha^−1^ in the control. The percentage difference in yield between the control and Variant 1 was 17% (*p* ≤ 0.05), whereas that between Variant 1 and Variant 2 was 16% (*p* ≤ 0.05).

Similarly, Roshni et al. [43] analyzed the effects of plant growth-promoting bacteria, including phosphate-solubilizing bacteria (PSB), potassium-solubilizing bacteria (KSB), *Azospirillum*, *Azotobacter*, and VAM (vesicular arbuscular mycorrhizae), combined with three chemical fertilization levels (100%, 75%, and 50% of the recommended dose of fertilizer (RDF), on carrot growth and yield. The experiment comprised 12 treatment combinations. The results showed that application of the full recommended dose of fertilizer (100% RDF = 75:60:50 kg ha^−1^) together with PSB + KSB + *Azospirillum* + *Azotobacter* + VAM resulted in significantly greater growth and yield than the other treatments. Among the interaction treatments, this combination resulted in the greatest plant height (72.59 cm), number of leaves (18.60), fresh and dry plant weights (107.57 g and 34.25 g, respectively), root length (23.07 cm), fresh and dry root weights (121.99 g and 37.52 g, respectively), harvest index (53.55%), yield per plot (5.82 kg plot^−1^), and yield per hectare (19.4 t ha^−1^). Compared with the treatment receiving only 100% RDF, carrot yield increased by 12% when all beneficial microorganisms were applied together with the full RDF.

Nikmatullah et al. [44] compared carrot growth and yield at three elevations (650, 550, and 175 m.a.s.l.) in plants inoculated with two biofertilizers: ExtraGen, containing *Pseudomonas*, *Bacillus megaterium*, *Azotobacter*, yeast, *Azospirillum*, *Actinomycetes*, *Lactobacillus*, and auxins, gibberellins, and cytokinins; and Bio-EXTRIM, containing PSB and VAM, *Azospirillum*, *Azotobacter*, and *Rhizobium*. The treatments also included chicken manure and two bioinsecticides containing *Metarhizium anisopliae* and *Beauveria bassiana*. The growth and yield of carrot plants were differentially influenced by biofertilizers at different land altitudes. At medium altitudes (650 and 550 m.a.s.l.), the application of the biofertilizer ExtraGen increased the carrot plant height, number of leaves, carrot yield, and carrot diameter. In lowland areas, the application of the biofertilizer Bio-EXTRIM did not influence plant height, the number of leaves, fresh or dry leaf biomass, root yield, or the length or diameter of carrot plants. Bio-EXTRIM increased the sweetness of carrot at low altitudes, but at medium and high altitudes, ExtraGen performed better. The carrot yield was greater in plants inoculated with ExtraGen than in the non-inoculated control plants at 550 m.a.s.l. (31,993.5 vs. 27,722.1 t ha^−1^; percentage increase = 15.4%) and 650 m.a.s.l. (24,327.0 vs. 21,473.0 t ha^−1^; percentage increase = 13.3%).

Sharmila et al. [45] evaluated the performance of several characteristics of two carrot varieties under different combinations of three growth-promoting microorganisms, namely, *Azospirillum*, VAM and PSB, when 50% of the recommended dose of chemical fertilization (RDF) was applied; these combinations were compared with the 100% RDF. Among the interaction effects, the combination of 50% RDF + PSB + *Azospirillum* + VAM had the highest values in terms of growth characteristics, namely, plant height (87.82 cm), number of leaves (19.74), fresh weight of roots (124.91 g) and dry weight of roots (8.84 g), yield characteristics (root length = 27.33 cm, yield per plot = 5.82 kg plot^−1^ and yield per hectare = 20.81 t ha^−1^), and carrot quality (carotene content = 47.39 mg). The net percentage increase in yield of this treatment over the 100% RDF treatment was 20.7% (i.e., 3.57 t ha^−1^; 20.81 vs. 17.24 t ha^−1^). The total soluble solids were higher with the application of *Azospirillum* and 50% RDF, whereas ascorbic acid was superior with the combination of *Azospirillum* + VAM + 50% RDF.

Zhu et al. [46] isolated and screened 12 high-efficiency phosphorus-solubilizing bacteria, one nitrogen-fixing strain, and two potassium-solubilizing strains from the carrot rhizosphere. These isolates included *Bacillus firmus* MN3 for nitrogen fixation, *Acinetobacter pittii* MP41 for phosphate solubilization, and *Bacillus subtilis* PK9 for potassium solubilization. These strains were used to formulate a microbial consortium for carrot inoculation and further analysis. Compared with the control, the application of the bacterial consortium significantly enhanced carrot growth, with increases in plant height of 17.1% and root length of 54.5% in a pot experiment. Furthermore, field trials confirmed the efficacy of the formulated bacterial consortium, with a notable 12.5% increase in carrot yield and minimal disturbance to soil bacterial diversity and abundance.

Gunasekara et al. [47] analyzed the effects of inoculating *Azotobacter* spp. and *Trichoderma asperellum* in plots with reduced rates of chemical fertilizers on the growth and yield characteristics of carrots. The results showed that the combined use of *Trichoderma asperellum* and *Azotobacter* spp. with reduced levels of (25% and 50%) nitrogen- and phosphorus-based inorganic fertilizers increased carrot growth and yield to levels equal to or greater than those obtained by using the full recommended dose of chemical fertilizer.

In the present study, the average yield increase across both carrot varieties following inoculation with conventional biological products compared with that in the non-inoculated control plants was 5.30 t ha^−1^ (14.0% increase). These values are within the expected increases in carrot yield already discussed when conventional biological products are applied. Zdravkovska et al. [42] reported a 17% increase in carrot yield following inoculation with *Azotobacter*, nitrifying microorganisms, and phosphate-solubilizing microorganisms. Roshni et al. [43] reported a 12% increase in carrot yield following inoculation with phosphate-solubilizing bacteria, potassium-solubilizing bacteria, *Azospirillum*, *Azotobacter*, and VAM under 100% RDF. Nikmatullah et al. [44] reported increases in the yield of carrots cultivated with chicken manure and those inoculated with a microbial consortium containing *Pseudomonas*, *Bacillus megaterium*, *Azotobacter*, yeast, *Azospirillum*, *Actinomycetes*, and *Lactobacillus* at two different altitudes: 15.4% at 550 m.a.s.l. and 13.3% at 650 m.a.s.l. Sharmila et al. [45], reported a 20.7% increase in carrot yield using PSB, *Azospirillum*, and VAM under 50% RDF. Zhu et al. [46] reported a 12.5% increase in carrot following inoculation with *Bacillus firmus* MN3, *Acinetobacter pittii*, and *Bacillus subtilis* PK9 under an optimized fertilization regime.

Although conventional biological products improved carrot yield relative to the non-inoculated control plants, the present results demonstrate the superior performance of systemic biological products. Compared with the control, systemic biological products increased the average carrot yield by 11.92 t ha^−1^ (32%). The average yield advantage of systemic over conventional biological products was 6.62 t ha^−1^ (15.4%). This additional increase in yield represents a greater potential economic return for farmers.

The findings of this study regarding improvements in carrot performance can be explained by the plant growth-promoting activities of the microorganisms contained in the biological products. Conventional biological products primarily act by modulating the rhizosphere, phyllosphere, or surrounding soil without colonizing internal plant tissues [48,49]. This aligns with the findings of the present study, as microorganisms from the conventional biological products were not detected within the internal tissues of carrot plants (Table 6). Their primary mode of action is therefore associated with modifications to the physical, chemical, and biological properties of the rhizosphere.

Direct mechanisms by which plant growth-promoting microorganisms enhance plant growth include nitrogen fixation, nutrient solubilization (phosphorus, potassium, and iron), and the production of growth regulators (auxins, gibberellins, and cytokinins). Indirect mechanisms include antibiosis and the production of antimicrobial metabolites; competition for iron, ecological niches, and carbon sources; hyperparasitism and predation; induction of systemic resistance; and interference with *quorum sensing* [50].

The mechanisms of action of the systemic biofertilizer used in this study include nitrogen fixation, phosphate solubilization, hormone production, ACC deaminase activity, and siderophore production [21]. Although conventional and systemic biological products may contain similar microbial species with comparable plant growth-promoting activities, the localization of these microorganisms results in significant differences in their effects on plant performance. In systemic biological products, all compounds involved in plant growth promotion, including hormones, nitrogen, ACC deaminase, antibiotics and volatile compounds, act more directly on plant physiology and biochemistry because they are released within internal plant tissues, avoiding the losses associated with microorganisms residing in the rhizosphere or surrounding soil [10,11,12].

The use of systemic bionematicide also clearly contributed to improved carrot performance because root damage was significantly lower in plants inoculated with this product. The mean damage across both varieties was 10.15% with the systemic nematicide, compared with 21.15% with the conventional nematicide and 31.6% in non-inoculated carrots. Because the nematode life cycle includes a stage within plant roots, improved control by microorganisms residing inside plant tissues would be expected. This is important because nematode damage in carrot plants can result in yield losses ranging from 20% to 70% [51,52].

Finally, analyses conducted of microorganisms reisolated from carrot plants confirmed the presence of the beneficial bacteria and fungi included in the two systemic biological products, although fungi were detected at relatively lower levels in both carrot varieties. Lin et al. [53] and Chen et al. [54] reported that fungal endophytes were less abundant than bacterial endophytes in plant stems. Different strategies used by bacteria and fungi to overcome stomatal closure and enter plant tissues have been described [55,56]. Based on our previous experience, bacteria appear to be more efficient than fungi at colonizing leaves through stomata [22].

The presence of endophytes in plants is influenced by various factors [57]. The frequency of plant colonization varies considerably and depends strongly on plant species, tissue type (with roots often showing relatively high colonization rates), environmental conditions (e.g., soil pH and stress), and season. Colonization frequencies range from low percentages (e.g., 3–13% tissue colonization in some tomato plants) to very high levels, depending on transmission efficiency (vertical vs. horizontal) and host-endophyte fitness trade-offs [53]. Some authors have reported that the abundance and diversity of endophytic microbial communities are significantly greater in organically farmed plants than in conventionally cultured plants [58].

The composition and diversity of endophytic communities are influenced by multiple factors, including soil chemistry [59], soil fertility, and climatic and environmental conditions at the cultivation site [60]. Additionally, host plant characteristics, including genotype, tissue type, age, and species, play significant roles [61]. Notably, the relative abundance of endophytes tends to increase as plants mature. Therefore, because carrots have a shorter growing cycle of approximately 3–4 months, they are generally expected to harbor few or no endophytes [62].

We expect systemic biological technologies to expand into a wider range of environments, crops, and regions, thereby increasing the productivity and quality of agricultural production. BIOqualitum is currently developing strategies to promote the use of this technology worldwide. Systemic biologicals are not merely a technology but rather a technological system that includes the production and formulation of systemic products, as well as the development of application strategies tailored to different crops (including timing, amount, mode, and frequency of application). Notably, products based on endophytes have been described as next-generation biologicals [63].

## 5. Conclusions

The results of this study showed that systemic biological products outperformed conventional biological products in promoting carrot growth and yield. Carrot yield increased by 11.92 t ha^−1^ (32%) in plants inoculated with systemic biological products compared with non-inoculated plants and by 6.62 t ha^−1^ (15.4%) compared with plants inoculated with conventional biological products. These increases in yield represent an important potential economic return for farmers. We expect this second-generation biological technology will be extended worldwide to boost agricultural food production.

## Figures and Tables

**Table 1:** Composition of the vehicle used for the formulation of the biological products employed in this study.

Component	%
Protein	9.3
Polysaccharides	8.2
Carbohydrates	9.3
Phosphorus	0.7
Potassium	1.2
Iron	1.9
Calcium	0.5
Magnesium	0.5

**Table 2:** Expected band sizes (bp) generated by the amplification of the ITS region and its restriction patterns (bp) obtained with the *DdeI* enzyme in the four bacteria.

Component	Bacterial Species			
	*P. fluorescens*	*A. brasilense*	*B. subtilis*	*B. mojavensis*
Amplified fragments from ITS region	692	668	455	233
	654		265	178
	614			
Restriction fragments generated with *DdeI*	195	190	384	178
	138	122	208	124
	105	88	239	

**Table 3:** Total chlorophyll content (mg g^−1^ FW) in carrot plants treated with conventional and systemic biological products.

Treatment	Variety	
	‘Romance’	‘Baltimore’
Control	10.8 a ^1^	11.1 a
Conventional	11.5 a	12.2 a
Systemic	17.3 b	16.9 b

^1^ Within each column, values followed by the same letter are not significantly different among the control (without biological products), conventional biological products, and systemic biological products for each carrot variety, as determined by Tukey’s multiple comparison test (*p* > 0.05).

**Table 4:** Growth variables and yields measured 120 days after seeding in two carrot varieties inoculated with conventional and systemic biological products in San Juan de la Vega, Guanajuato, Mexico, in 2021.

Carrot Variety	Root Diameter (cm)			Plant Length (cm)			No. Leaves			Root Weight (g)			Yield (t ha^−1^)		
		Inoculated			Inoculated			Inoculated			Inoculated			Inoculated	
	Control	Conv.	Syst.	Control	Conv.	Syst.	Control	Conv.	Syst.	Control	Conv.	Syst.	Control	Conv.	Syst.
‘Romance’	2.78 a ^1^	3.07 b	3.24 c	36.55 a	38.00 a	39.80 b	9.62 a	9.93 a	10.58 b	111.35 a	122.71 b	145.00 c	37.40 a	43.23 b	49.10 c
Increase (%)	-	10.6	16.7	-	4.0	8.9	-	3.2	10.0	-	10.2	30.2	-	15.6	31.3
‘Baltimore’	2.84 a	3.12 b	3.63 c	39.30 a	42.68 a	47.40 b	10.30 a	11.30 b	12.70 b	116.90 a	130.11 b	151.30 c	38.03 a	42.80 b	50.16 c
Increase (%)	-	11.5	27.8	-	8.6	20.6	-	9.7	23.3	-	11.3	29.4	-	12.5	31.9

^1^ Within each row, values followed by the same letter are not significantly different among the control (without biological products), conventional biological products (Conv.), and systemic biological products (Syst.), as determined by Tukey’s multiple comparison test (*p* > 0.05).

**Table 5:** Percentage of carrot roots damaged by nematodes in two varieties inoculated with conventional or systemic biological products.

Treatment	Variety	
	‘Romance’	‘Baltimore’
Control	32.9 a ^1^	30.3 a
Conventional	22.1 b	20.2 b
Systemic	11.0 c	9.3 c

^1^ With each column, values followed by the same letter are not significantly different among the control (without biological products), conventional biological products, and systemic biological products for each carrot variety, as determined by Fisher’s exact test (*p* > 0.05).

**Table 6:** Presence (%) of beneficial microorganisms within the internal tissues of carrot plants inoculated with systemic or conventional biological products.

Microorganism	Product	Variety	
		‘Romance’	‘Baltimore’
*Pseudomonas fluorescens* Ag_001	Systemic	95	90
*Azospirillum brasilense* Ag_001	Systemic	91	87
*Bacillus subtilis* Ag_001	Systemic	90	86
*Bacillus mojavensis* B3-15	Systemic	92	88
*Paecilomyces lilacinus* Ag_001	Systemic	42	43
*Pochonia chlamydosporia* Ag_001	Systemic	38	45
*Pseudomonas fluorescens*	Conventional	0	0
*Azospirillum brasilense*	Conventional	0	0
*Bacillus subtilis*	Conventional	0	0
*Bacillus mojavensis*	Conventional	0	0
*Paecilomyces lilacinus*	Conventional	0	0
*Pochonia chlamydosporia*	Conventional	0	0

## Data Availability

Data are available on request from the corresponding authors.
